# Impact of Cannabidiol and Exercise on Clinical Outcomes and Gut Microbiota for Chemotherapy-Induced Peripheral Neuropathy in Cancer Survivors: A Case Report

**DOI:** 10.3390/ph17070834

**Published:** 2024-06-25

**Authors:** MariaLuisa Vigano, Sarah Kubal, Yao Lu, Sarah Habib, Suzanne Samarani, Georgina Cama, Charles Viau, Houman Farzin, Nebras Koudieh, Jianguo Xia, Ali Ahmad, Antonio Vigano, Cecilia T. Costiniuk

**Affiliations:** 1 Division of Experimental Medicine, Faculty of Medicine and Health Sciences, McGill University, Montreal, QC H3A 0G4, Canada; 2 Infectious Diseases and Immunity in Global Health Program, Research Institute of the McGill University Health Centre, Montreal, QC H4A 3J1, Canada; 3 Division of Supportive and Palliative Care, McGill University Health Centre, Montreal, QC H4A 3J1, Canada; 4 Institute of Parasitology, Faculty of Agricultural and Environmental Sciences, McGill University, Ste-Anne-de-Bellevue, QC H9X 3V9, Canada; 5 Division of Palliative Care, Jewish General Hospital, Montreal, QC H3T 1E2, Canada; 6 Department of Microbiology and Immunology, McGill University, Montreal, QC H3A 0G4, Canada; 7 Division of Infectious Diseases and Chronic Viral Illness Service, Royal Victoria Hospital—Glen Site, McGill University Health Centre, Montreal, QC H4A 3J1, Canada

**Keywords:** gut microbiota, chemotherapy-induced peripheral neuropathy, neuropathic pain, cannabidiol, exercise, survivorship

## Abstract

Chemotherapy-induced peripheral neuropathy (CIPN) remains a clinical challenge for up to 80% of breast cancer survivors. In an open-label study, participants underwent three interventions: standard care (duloxetine) for 1 month (Phase 1), oral cannabidiol (CBD) for 2 months (Phase 2), and CBD plus multi-modal exercise (MME) for another 2 months (Phase 3). Clinical outcomes and gut microbiota composition were assessed at baseline and after each phase. We present the case of a 52-year-old female with a history of triple-negative breast cancer in remission for over five years presenting with CIPN. She showed decreased monocyte counts, c-reactive protein, and systemic inflammatory index after each phase. Duloxetine provided moderate benefits and intolerable side effects (hyperhidrosis). She experienced the best improvement and least side effects with the combined (CBD plus MME) phase. Noteworthy were clinically meaningful improvements in CIPN symptoms, quality of life (QoL), and perceived physical function, as well as improvements in pain, mobility, hand/finger dexterity, and upper and lower body strength. CBD and MME altered gut microbiota, showing enrichment of genera that produce short-chain fatty acids. CBD and MME may improve CIPN symptoms, QoL, and physical function through anti-inflammatory and neuroprotective effects in cancer survivors suffering from long-standing CIPN.

## 1. Introduction

Despite improvements in survivorship rates, chemotherapy-induced peripheral neuropathy (CIPN) remains a side effect in up to 80% of breast cancer survivors who received taxane- and/or platinum-based chemotherapy [1,2,3]. CIPN is primarily a distal nerve syndrome caused by damage and inflammation [4,5,6]. Due to pain and sensory impairment, CIPN is characterized by reduced functional status and quality of life (QoL) [1,2,3]. Currently, there are no effective treatments available for CIPN, as conventional options such as opioids and serotonin–norepinephrine reuptake inhibitors like duloxetine provide limited benefits and are plagued by side effects [7,8]. Hence, there is an urgent need to discover more effective and safer therapies for CIPN.

Cannabidiol (CBD) and multi-modal exercise (MME) are promising therapies due to their anti-inflammatory and analgesic effects. The main non-psychoactive ingredient of Cannabis sativa, CBD, exerts its biological effects as an inverse and non-competitive allosteric agonist of the cannabinoid receptor 1 (CB1), with little or no affinity for cannabinoid receptor 2 (CB2) [9]. Furthermore, it also boosts the endocannabinoid system (ECS) through other non-canonical receptors, such as GPR55 and Transient Receptor Potential (TRP) channels [9]. The ECS is involved in physiological processes like immunomodulation and analgesia. In the context of CIPN, CBD was shown to attenuate chemotherapy-induced mechanical allodynia in mice [10]. CBD competes with endocannabinoids for the binding to certain intracellular fatty acid binding proteins (FABP-3, -5, and -7) and prevents their degradation through fatty acid amide hydrolase (FAAH) [11]. In another preclinical model, the antiallodynic and anxiolytic effects of CBD were completely blocked by a TRPV-1 (a TRP of the vanilloid family) antagonist (Capsazepine) and partially inhibited by 5-HT_1A_ receptor antagonist (Way 100635) [12]. These observations may explain why in a clinical study on CIPN, CBD was shown to reduce numbness and tingling but not pain as compared to placebo [13].

Low to moderate-intensity aerobic and resistance exercises may relieve CIPN symptoms by improving the vascular function and metabolic activity of peripheral nerves, up-regulating neurotrophic factors and reducing inflammation [4,14,15]. Recent studies have suggested a crosstalk between exercise and ECS [16]. A decreased levels of endocannabinoids have been reported in the peripheral nervous system in animal models of CIPN [17]. Exercise has been shown to increase the levels of circulating endocannabinoids [16,18]. We hypothesized that combining CBD and exercise may exert additive beneficial effects on CIPN symptoms. Furthermore, an extensive link between gut microbiota and both endocannabinoids and exercise has been well documented in vivo [19,20,21,22]. We anticipated that CBD and MME would also induce host beneficial changes in the gut microbiota composition.

**Figure 1:**
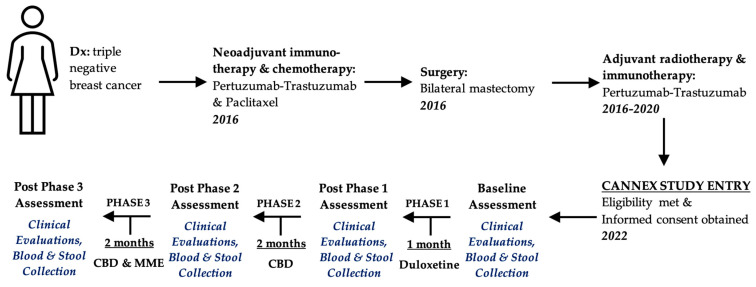
Patient’s trajectory. Timings are shown for the collection of samples (peripheral blood and feces), completion of self-reported questionnaires (FACT-GOG-Ntx scales, painDETECT, Godin-Shepard leisure-time physical activity), and various functional tests for mobility, strength, and hand/finger dexterity. Blood samples were analyzed for immune parameters, and the DNA from the fecal samples was sequenced to determine the V4 of the 16S rRNA gene.

To test our hypothesis, a prospective, open-label study (CANNEX: Cannabis-Exercise study, MUHC REB 2022-8570) was conducted in cancer survivors suffering from CIPN at the McGill University Health Centre (MUHC). After providing written informed consent, patients underwent three interventions as described in Figure 1. In Phase 1, participants received up to 60 mg/day of duloxetine for one month (standard treatment). In Phase 2, participants who did not respond and/or could not tolerate the side effects of duloxetine were offered a CBD isolate (Clear Oil, Spectrum Therapeutics, Smith Falls, ON, Canada). The CBD isolate was administered orally; the dose was gradually titrated up to 300 mg/day (starting dose 20 mg/day) as tolerated. In Phase 3, these same participants were offered over another two months a combined therapy: CBD and a multi-modal exercise (MME) program comprising moderate-intensity aerobic, resistance (a combination of calisthenics and Therabands^®^), and balance components. The intensity of aerobic and resistance exercises was gauged using both the Karvonen target heart rate method and the Borg 6–20 rating of perceived exertion scale. The MME program was completed at least three times weekly, including one supervised exercise session with a kinesiologist per week. Progressions in the exercise program were administered as required and according to perceived exertion during the supervised sessions. The objectives of the study were to determine the safety and effectiveness of oral CBD and MME for improving functional status, QoL, CIPN-associated symptoms, inflammatory markers and for causing host-beneficial changes in the gut microbiota composition.

Participants were assessed for: (a) pain characteristics using the painDETECT questionnaire [23,24], (b) physical activity using Godin-Shephard leisure-time physical activity questionnaire (GSLPAQ) [25], (c) functional capacity using the gait speed (GS) test for mobility, the 9-hole peg (9-HPT) test for finger/hand dexterity, 5 times Sit to Stand (5× STS) for lower body strength and hand grip strength (HGS) for upper body strength; (d) appendicular skeletal muscle index (ASMI) via dual-energy X-ray absorptiometry (DEXA); (e) CIPN symptomatology, perceived physical function, and overall QoL via Functional Assessment of Cancer Therapy/Gynecologic Oncology Group-Neurotoxicity (FACT-GOG-Ntx-13 v.4) questionnaire (five domains considered: physical, functional, social, and emotional well-being and CIPN symptomatology) [26]. The CIPN symptomatology subscale (FACT-GOG-Ntx) can act as a marker alone for CIPN-related neurotoxic symptoms, as it assesses the sensory, motor, auditory and cold sensitivity problems experienced with this condition [26]. Perceived physical function was assessed through the FACT-GOG-Ntx Trial Outcome Index (FACT-GOG-Ntx TOI), which takes into consideration three domains of health-related QoL: physical well-being, functional well-being, and CIPN symptomatology [26]. The overall QoL (FACT-GOG Total) score evaluated four of the five domains (physical, functional, social, and emotional well-being, 0–108) [26]. Higher scores for FACT-GOG-Ntx-13 v.4 and lower scores for the painDETECT questionnaires indicate improvement [23,24,27,28]. Clinically meaningful improvements were considered: 3.68 points increases for CIPN symptoms (FACT-GOG-Ntx), 6 for perceived physical function (FACT-GOG-Ntx TOI), and 7 for overall QoL (FACT-GOG Total) [28,29]. At baseline and the end of each phase, non-fasting blood and fecal samples were collected for inflammatory markers (Table 1) and microbiota investigations, respectively. The taxa-abundance profiling of fecal microbiota was performed after 16S rRNA gene (V4 region) sequencing using the Illumina MiSeq platform and analyzed using the DADA2 pipeline and Silva database on MicrobiomeAnalyst 2.0 [30]. Potential diet changes for each study participant were also monitored through a free web-based Automated Self-Administered 24-Hour Dietary Assessment Tool [31].

## 2. Case Presentation

A 52-year-old Caucasian woman had a history of stage IV (presence of solitary liver metastasis, which disappeared after neoadjuvant chemotherapy) triple-negative breast cancer in remission for over five years (Figure 1). She presented with CIPN in her feet and neuropathic pain around her right axilla and shoulder. She had previously received neoadjuvant chemotherapy and immunotherapy between 26 January 2016 and 19 April 2016 in the form of four cycles of paclitaxel and two anti-HER2 monoclonal antibodies (trastuzumab–pertuzumab). She then underwent a bilateral mastectomy and left axillary lymph node dissection, followed by adjuvant immunotherapy and radiotherapy between 19 April 2016 and 14 July 2020. Immunotherapy consisted of 74 cycles of trastuzumab–pertuzumab, and 40.05 Gy of radiation was delivered in 15 fractions to the whole left breast, supraclavicular, and internal mammary nodes. Her comorbidities included liver steatosis, osteoarthrosis, and lymphedema.

The anthropometric and biologic parameters of the patient are shown in Table 1.

At baseline (BL), the patient’s PBMC count, CRP count, and absolute monocyte count were moderately elevated, but her lymphocyte percentage was normal (Table 1). The woman was moderately active and her painDETECT was consistent with a neuropathic type of pain (Table 2). 

**Figure 2:**
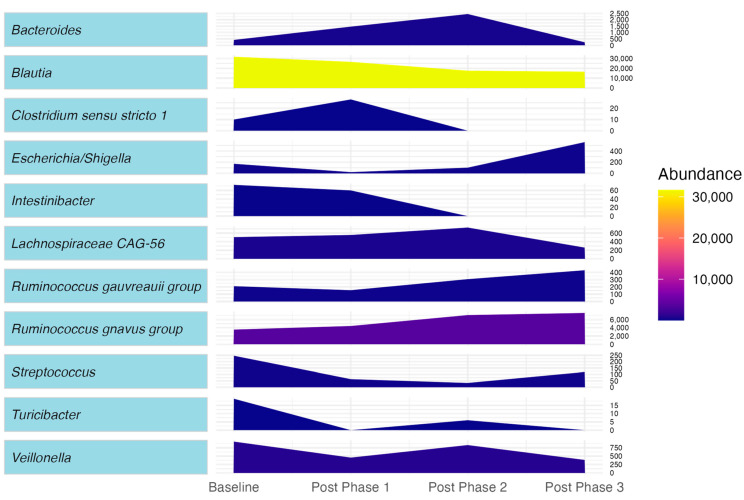
Changes in microbiota genera abundance across different study time points.

The patient’s microbiota was enriched in *Blautia*, *Streptococcus*, *Intestinibacter*, and *Turicibacter* and least abundant in *Ruminococcus gnavus* and *Bacteroides* (Figure 2).

During Phase 1, the patient took duloxetine 30 mg/day for only one week as she developed intolerable hyperhidrosis. Nevertheless, we observed small improvements in some of her clinical measures. Compared with BL, her relative lymphocyte percentage became elevated, while her PBMC count normalized (Table 1). All measures of systemic inflammation (SII, PLR, MLR, and NLR) decreased. Her level of physical activity changed to active. Her painDETECT improved by 11% (Table 2). Her CIPN symptoms (FACT-GOG-Ntx) and perceived physical function (FACT-GOG-Ntx TOI) did not improve. However, she experienced a clinically meaningful improvement in her overall QoL (10 points increase on the FACT-GOG Total). Functional tests improved by 10% in mobility (GS test), 33% in lower body strength (5× STS), 4% in upper body strength (HGS) and 8% in finger/hand dexterity (9-HPT) in comparison to BL (Table 2). Taxa profiling revealed an abundance of *Clostridium sensu stricto 1* and *Intestinibacter* belonging to the Firmicutes phylum and the least abundance in *Escherichia*-*Shigella*, *Ruminococcus gauvreauii*, *Veillonella*, and *Turicibacter* (Figure 2).

During Phase 2, CBD administration resulted in better clinical outcomes as compared to BL and Phase 1. The patient reached the maximal dose of CBD (300 mg/day). She only reported mild, self-resolving diarrhea while transitioning from 100 mg to 200 mg of daily CBD. Her liver enzymes remained within normal limits. All previously elevated parameters (CRP, %lymphocytes) normalized, except for the PBMC count (Table 1). All measures of systemic inflammation (SII, PLR, MLR, and NLR) decreased as compared to BL. The participant was more active (45% increase in GSLPAQ score from BL) (Table 2). She experienced clinically a meaningful improvement in perceived physical function (10 points increase on the FACT-GOG-Ntx TOI) and CIPN symptoms (7 points increase on the FACT-GOG-Ntx) as compared to BL. Her painDETECT decreased by one point from BL. Functional tests revealed improvements of 19% in mobility (GS test), 24% in lower body strength (5× STS), 7% in upper body strength (HGS), and 5% in finger/hand dexterity (9-HPT) as compared to BL. Taxa profiling revealed the lowest Firmicutes to Bacteroidetes (F/B) ratio. Propionate-producing genera *Veillonella*, *Bacteroides*, *Lachnospiraceae CAG-56*, and butyrate-producing genus *Turicibacter* [32,33,34] were enriched. The least abundant taxa included *Blautia*, *Streptococcus*, *Intestinibacter*, and *Clostridium sensu stricto 1* (Figure 2).

During Phase 3, clinical and biological outcomes showed the best improvement. The patient continued taking 300 mg/day of CBD while undergoing an 8-week MME program. No intervention-related adverse effects were noted during this phase. Her blood parameters and liver enzymes remained within normal limits except for the PBMC count, which was still elevated but slightly decreased in comparison to Phase 2 (Table 1). All measures of systemic inflammation (SII, PLR, MLR, and NLR) decreased in comparison to both BL and Phase 2. She was more active (52% increase in GSLPAQ score from BL) (Table 2). She experienced clinically meaningful improvements in perceived physical function (19 points increase on the FACT-GOG-Ntx TOI), overall QoL (14.3 points increase on the FACT-GOG Total), and CIPN symptoms (11 points increase on the FACT-GOG-Ntx) as compared to BL (Table 2). Her painDETECT showed a 33% improvement from BL (Table 2). She also experienced improvements in mobility (27%, GS test), lower body strength (27%, 5× STS) and upper body strength (11%, HGS) and finger/hand dexterity (17%, 9-HPT) as compared to BL (Table 2). There was also an increase in *Ruminococcus gnavus*, *Ruminococcus gauvreauii*, and *Escherichia/Shigella* genera in her feces. Three genera, *Clostridium sensu stricto 1*, *Turicibacter*, and *Intestinibacter* genera, were decreased in her microbiota (Figure 2). The patient reported no changes in her eating habits during all study phases.

## 3. Discussion

There is no gold standard for treating CIPN; the only FDA-approved medication for CIPN is duloxetine. The patient showed improvement in the functional measures, QoL and pain with duloxetine (Phase 1). However, intolerable side effects did not allow for the continuation of this treatment. Our findings are consistent with the reported challenges for duloxetine use in CIPN: the majority of patients treated with this medication experience moderate benefits, which are often associated with a number of side effects, such as nausea, fatigue, and dizziness [7,8,35].

CBD appeared to be a better alternative than duloxetine for CIPN. Its administration (Phase 2) was associated with an overall improvement in the functional measures and clinically meaningful improvements in CIPN symptoms and perceived physical function. Despite our patient was suffering from liver steatosis, regular CBD doses of 300 mg/day did not cause any alteration in her liver function tests. 

The therapeutic role of cannabinoids for peripheral neuropathy has been previously explored for diabetes, HIV-related neuropathy, and, more recently, for taxane-related CIPN [36,37]. To our knowledge, only three studies (one pilot study, one retrospective analysis, and one observational study) have assessed the clinical effect of medicinal cannabis (MC) in CIPN [38,39]. The 16-patient double-blind, placebo-controlled, crossover pilot study by Lynch et al. examined nabiximols (a balanced formulation of THC and CBD to be administered sublingually) and reported no statistically significant effect compared to placebo [38]. The retrospective analysis by Waissengrin et al. found a reduced rate of CIPN in patients who took MC, suggesting a protective effect [39]. Finally, a recent study by Nielsen et al. demonstrated that oral CBD (300 mg/day) given to patients prior to chemotherapy administration attenuated early CIPN symptoms with no major safety concerns [40]. While our patient showed improvement in CIPN symptoms and functional status, her painDETECT score remained close to BL after Phase 2. A systemic review and meta-analysis of eighteen placebo-controlled studies found that for healthy individuals undergoing experimental pain, cannabinoids may not reduce the intensity of pain but rather increase tolerability and reduce the perceived unpleasantness of painful stimuli [41]. 

In this phase, CBD also appeared to alter the gut microbiota composition of the patient. The Firmicutes/Bacteroidetes (F/B) ratio was the lowest, and propionate and butyrate-producing genera such as *Veillonella*, *Bacteroides*, *Lachnospiraceae CAG-56*, and *Turicibacter* were increased. This result is consistent with a previous study in mice, which found a decreased F/B ratio with cannabinoid administration [42]. Propionate is one of the three main short-chain fatty acids (SCFAs) that regulate gut homeostasis [43] and exerts neuroprotective and neurogenerative effects on Schwann cells and dorsal root ganglia in the peripheral nervous system. There is also clinical evidence of propionate’s immunoregulatory effect in multiple sclerosis patients [44,45,46]. *Bacteroides*, the largest contributors to propionate formation in the gut, have been shown to play a pivotal role in immunomodulation and protection from inflammatory arthritis. This genus is less abundant in the microbiota of ulcerative colitis, inflammatory bowel disease and diabetic patients who also suffer from peripheral neuropathy [47,48,49,50]. In murine models, butyrate, another main SCFA, was shown to prevent morphine and paclitaxel-induced nociceptive hypersensitivity and act as an anti-inflammatory agent [51,52,53]. Additionally, secondary to CBD administration, a decrease in genera such as *Blautia*, *Streptococcus*, *Intestinibacter*, and *Clostridium sensu stricto 1* was observed. Of these, gut *Streptococcus* spp. has been associated with circulating biomarkers of systemic inflammation [54]. The *Blautia* genus was found to be abundant in diabetic patients suffering from peripheral neuropathy whereas *Clostridium sensu stricto 1* and *Intestinibacter* were increased in osteoporosis patients [48,55]. Our findings suggest that CBD is associated with a positive/healthier shift in the gut microbial composition. 

The combined CBD and multi-modal exercise (MME) intervention (Phase 3) was most beneficial and well tolerated. Our patient experienced not only improvement in all functional measures and the largest reduction in pain score but also showed clinically meaningful improvements in CIPN symptoms, perceived physical function and overall QoL. MME has been previously suggested to be a feasible and effective treatment for CIPN [4,15,56,57]. The therapeutic crosstalk of cannabinoids and physical exercise has been linked to increase serum concentrations of endocannabinoids [16]. During exercise, the endocannabinoid system stimulates the peripheral nervous system via Transient Receptor Potential (TRP) channels; this mechanism activates the cholinergic anti-inflammatory pathway and reduces chronic inflammation [17]. CBD has been suggested to prevent the degradation of endocannabinoids [11]. It acts as an inverse allosteric modulator of CB1, has little or no affinity for CB2, can interact with several non-canonical endocannabinoid receptors, such as TRP channels and the 5-hydroxytryptamine (5-HT; serotonin) receptors 5-HT_1A_ and 5-HT2_A_, and regulates functional activities of several neurotransmitters in the brain and peripheral nervous system [9]. 

Treatment with CBD plus MME also altered the patient’s intestinal microbiota toward a more “anti-inflammatory” profile. Three genera were found to be most abundant: *Ruminococcus gnavus*, *Ruminococcus gauvreauii*, and *Escherichia/Shigella*. *Ruminococcus gnavus* and *Escherichia/Shigella* both produce serotonin in mice via tryptamine and propionate ester. Spohn et al. suggests that gut-derived 5-HT exerts anti-inflammatory 5-HT_4_ receptor-mediated effects under normal conditions and pro-inflammatory 5-HT_7_ receptor-mediated actions under pathological conditions [58]. Furthermore, CBD and MME may alleviate CIPN symptoms and improve QoL by promoting SCFAs- and serotonin-producing genera in the gut, as it was observed in murine models [50,51,52].

The study has certain limitations. This case report examines the outcomes of one individual only. Nevertheless, a cursory look at other patients’ results in our study showed similar benefits from CBD and MME (unpublished data). As the study was open-label, a placebo effect cannot be ruled out. However, the positive changes in inflammatory markers and in the intestinal microbiota are less likely to be the result of a placebo effect. Our results should be verified using a larger cohort of CIPN patients, preferably in randomized, placebo-controlled clinical trials. The case is reported here solely for the purpose of information. We in no way imply, suggest, or recommend using CBD with or without exercise to cancer patients/survivors for the treatment of CIPN. The decision should be made by their physicians.

## 4. Conclusions

This case report provides initial but holistic evidence supporting complementary approaches for addressing CIPN in cancer survivors. It suggests that clinically meaningful improvements in CIPN symptoms, quality of life, and functional status can be achieved through combining the oral administration of 300 mg/day of CBD with a multi-modal exercise program. The synergistic benefit of this combination may be explained through an increase in circulating endocannabinoids and beneficial changes in the gut microbiota. Both CBD and exercise were also found to be better tolerated than duloxetine.

## Figures and Tables

**Table 1:** Anthropometric and biologic parameters.

Variable (Unit; Normal Range)	Baseline	Post Phase 1	Post Phase 2	Post Phase 3
Weight (kg)	92.1	92.7	93.7	95.3
Height (cm)	166	166	166	166
BMI (kg/m^2^)	33.43	33.65	34.01	34.59
ASMI (kg/m^2^)	7.40	7.37	7.22	7.46
PBMC Count (10^6^/mL; 0.5–3)	3.1 *	2.8	5.4 *	4.4 *
Lymphocytes (%; 22–44)	32.53	45.20 *	38.48	37.27
Monocytes (%; 3–10)	12.69 *	9.23	9.78	8.64
Monocytes (absolute counts; 10^9^/L; 0–0.8)	0.84 *	0.53	0.58	0.53
CRP (mg/L; 0–5)	14.00 *	3.60	3.80	4
SII	420.28	206.68	323.96	334.19
PLR	123.61	88.33	111.71	107.46
MLR	0.39	0.21	0.26	0.23
NLR	1.57	0.91	1.31	1.36
Bilirubin (total; μmol/L; 1.7–18.9)	7.9	6.4	5.7	6.6
Alkaline phosphatase (U/L; 42–98)	68	60	68	62

ASMI: Appendicular skeletal muscle index; BMI: Body mass index; CRP: C-reactive protein; MLR: Monocyte to lymphocyte ratio; NLR: Neutrophil to lymphocyte ratio; PBMC: Peripheral blood mononuclear cells; PLR: Platelet to lymphocyte ratio; SII: Systemic Inflammation-Immune Index. * Values elevated than the upper limit of the normal range.

**Table 2:** Patient scores for the assessment of CIPN and physical performance.

Variable/Measure (Unit)	Baseline	Post Phase 1	Post Phase 2	Post Phase 3
PainDETECT Score	27	24	26	18
FACT-GOG-Ntx Score	37	34	44	48
FACT-GOG-Ntx TOI Score	74	78	84	93
FACT-GOG Total Score	67	77	73	81.3
GSLPA Score	22	30	40	46
GS (m/s)	0.91	1.00	1.08	1.16
5× STS (seconds)	14.65	9.8	11.09	10.69
HGS (kg)	27	28	29	30
9-HPT (seconds)	19.3	17.8	18.34	16

FACT-GOG-Ntx: Functional Assessment of Cancer Therapy/Gynecologic Oncology Group-Neurotoxicity (CIPN symptoms) subscale; FACT-GOG-Ntx TOI: FACT-GOG-Ntx Trial Outcome Index (perceived physical function, pPF); GSLPA: Godin-Shephard leisure-time physical activity; GS: Gait speed (mobility); 5× STS: 5 times Sit to Stand (lower body strength); HGS: hand grip strength (handheld dynamometer, upper body strength); 9-HPT: 9-Hole Peg Test (finger/hand dexterity).

## Data Availability

Data are contained within the article.
